# Function and Transcriptional Regulation of Bovine *TORC2* Gene in Adipocytes: Roles of *C/EBPγ*, *XBP1*, *INSM1* and *ZNF263*

**DOI:** 10.3390/ijms20184338

**Published:** 2019-09-04

**Authors:** Rajwali Khan, Sayed Haidar Abbas Raza, Zainaguli Junjvlieke, Wang Xiaoyu, Matthew Garcia, Ibrahim Elsaeid Elnour, Wang Hongbao, Zan Linsen

**Affiliations:** 1 College of Animal Science and Technology, Northwest A&F University, Yangling 712100, China (R.K.) (S.H.A.R.) (Z.J.) (W.X.) (I.E.E.) (W.H.B.); 2 Department of Livestock Management, Breeding and Genetics, The University of Agriculture, Peshawar 25130, Pakistan; 3 School of Animal Dairy and Veterinary Sciences, Utah State University, Logan, UT 84322, USA; 4 National Beef Cattle Improvement Research Center, Northwest A&F University, Yangling 712100, China

**Keywords:** TORC2, adipogenesis, preadipocytes proliferation and differentiation, transcription factors, luciferase reporter assay, bovine adipocytes, nuclear protein, intramuscular fat, DNA-Protein interaction, gene regulation

## Abstract

The *TORC2* gene is a member of the transducer of the regulated cyclic adenosine monophosphate (cAMP) response element binding protein gene family, which plays a key role in metabolism and adipogenesis. In the present study, we confirmed the role of *TORC2* in bovine preadipocyte proliferation through cell cycle staining flow cytometry, cell counting assay, 5-ethynyl-2′-deoxyuridine staining (EdU), and mRNA and protein expression analysis of proliferation-related marker genes. In addition, Oil red O staining analysis, immunofluorescence of adiponectin, mRNA and protein level expression of lipid related marker genes confirmed the role of *TORC2* in the regulation of bovine adipocyte differentiation. Furthermore, the transcription start site and sub-cellular localization of the *TORC2* gene was identified in bovine adipocytes. To investigate the underlying regulatory mechanism of the bovine *TORC2*, we cloned a 1990 bp of the 5’ untranslated region (5′UTR) promoter region into a luciferase reporter vector and seven vector fragments were constructed through serial deletion of the 5′UTR flanking region. The core promoter region of the *TORC2* gene was identified at location −314 to −69 bp upstream of the transcription start site. Based on the results of the transcriptional activities of the promoter vector fragments, luciferase activities of mutated fragments and siRNAs interference, four transcription factors (*CCAAT/enhancer-binding protein C/BEPγ, X-box binding protein 1 XBP1, Insulinoma-associated 1 INSM1,* and *Zinc finger protein 263 ZNF263*) were identified as the transcriptional regulators of *TORC2* gene. These findings were further confirmed through Electrophoretic Mobility Shift Assay (EMSA) within nuclear extracts of bovine adipocytes. Furthermore, we also identified that *C/EBPγ, XBP1, INSM1* and *ZNF263* regulate *TORC2* gene as activators in the promoter region. We can conclude that *TORC2* gene is potentially a positive regulator of adipogenesis. These findings will not only provide an insight for the improvement of intramuscular fat in cattle, but will enhance our understanding regarding therapeutic intervention of metabolic syndrome and obesity in public health as well.

## 1. Introduction

In production animals, adipose tissue plays a crucial function in the determination of meat sensory quality [1]. In the mammalian body, the main adipose depots are subcutaneous, visceral, intramuscular and intermuscular fat. Intramuscular fat is the most important factor that defines meat quality; however enhancing its deposition without increasing the other three adipose depots represents a challenge for the meat industry [2]. Adipogenesis is a very complex biological process under strict genetic control and is supported by hormones, enzymes, and metabolites that regulate cellular metabolism [3]. The deposition of intramuscular fat involves a continuous chain of processes that includes preadipocyte proliferation, differentiation and maturation [4,5]. Intramuscular fat (IMF) is composed of intramuscular adipocytes, and IMF content is one of the most important traits of meat quality, as it can significantly improve meat eating qualities. Highly marbled meat is very desirable; hence, methods to increase IMF content have become a key aspect of improving meat quality. Therefore, research on the mechanism of adipogenesis provides invaluable information for the improvement of meat quality [6]. Enzymes, such as adipose triglyceride lipase (ATGL), monoglyceride lipase (MAGL) and hormone sensitive lipase (HSL) regulate the deposition of intramuscular fat in cattle. The reduction of ATGL and MAGL lipolytic activities promotes intramuscular fat deposition in beef cattle [7]. Triacylglycerol (TAG) is hydrolyzed by the activities of these three enzymes [8]; however, catecholamines are vital for TAG hydrolysis as a supra-regulator, as it binds to β-adrenergic receptors in white adipocytes and subsequently stimulates adenyl cyclase, which catalyzes the production of cyclic AMP (cAMP). The increased concentration of cAMP stimulates protein kinase A (PKA)—which ultimately activates HSL [9]. Furthermore, the stimulated PKA activates ATGL [10]. *Transducer of regulated cAMP response element-binding protein (CREB) 2* (*TORC2*) is one of the main inhibitors of lipolysis by regulating PKA and HSL activities [11,12]. *TORC2* performs various roles in anabolic processes of adipocytes including lipogenesis, adipogenesis and lipids esterification [13]. Furthermore, dephosphorylation of the *TORC2* gene induces the overexpression of glucogenic genes including glucose-6-phosphatase, phosphoenolpyruvate carboxykinase and an increased rate of glucose production which in turn causes inhibition of lipase activity [14,15]. Therefore, the studies cited previously confirmed the role of *TORC2* gene in adipogenesis via the inhibition of cAMP/PKA lipolysis pathway.

There are three members of this gene family: *TORC1*, *TORC2* and *TORC3*. The *TORC* gene family is also known as CRTC [CREB (cAMP response element binding protein)-regulated transcription coactivator]. The *TORC2* gene is responsible for nutrient metabolism, gluconeogenesis, myogenesis and adipogenesis through the phosphoinositide 3-kinase-Akt (PI3K-Akt), adenosine monophosphate-activated protein kinase (AMPK), glucagon and insulin resistance signaling pathways via promotion of the anabolic and inhibiting catabolic processes within the cells [16]. Moreover, *TORC2* is a coactivator gene that plays a key role in glucagon-mediated activation of gluconeogenesis through a synchronized mechanism of glucocorticoid receptor and glucagon-CREB pathways coordinated with *PEPCK* and *G6P* genes [17,18,19,20,21]. Additionally, through the CREB pathway in coordination with peroxisome proliferator-activated receptor γ (*PPARγ*), coactivator 1α (*PGC1α*) and nuclear receptor subfamily 4 group A (*NR4A*), *TORC2* promotes gluconeogenesis and adipogenesis [22].

Moreover, lipogenesis and gluconeogenesis are well known contributors to the paradoxical effects of insulin resistance [23,24,25]. Lipogenesis is regulated by transcription factors including peroxisome proliferator-activated receptor gamma (PPARγ), carbohydrate response element binding protein (ChREBP), and sterol regulatory element-binding protein-1c (SREBP-1c), while gluconeogenesis is regulated by PPARγ, forkhead box protein O1 (FOXO1), PPARγ coactivator-1α (PGC-1α), TORC2 and CREB [9,26]. PPARγ is highly enriched in adipose tissue, where it performs a crucial role in insulin sensitivity, adipocyte differentiation and adipokine/cytokine secretion [27,28]. However, PPARγ transcription is regulated by co-activators including PGC-1α, and *TORC2* [29,30]. Therefore, we hypothesize that *TORC2* may perform a crucial role in the regulation of bovine adipogenesis. Intramuscular fat (marbling) is one of the most important indicators of meat quality grading. Unfortunately, molecular mechanisms regulating the bovine intramuscular fat through *TORC2* gene is still unexplained. Therefore, the present study was conducted to explore molecular function and regulatory mechanisms of *TORC2* gene in bovine adipocytes.

## 2. Results

### 2.1. Transfection Efficiency, Tissues and Cellular Expression of TORC2 Gene

**Figure 1:**
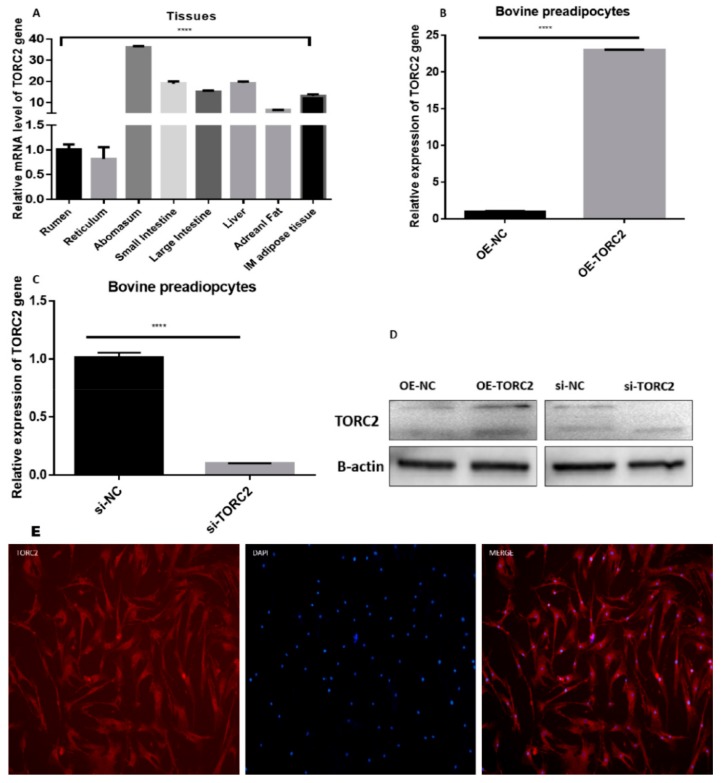
Transfection efficiency, tissue and cellular expression and sub cellular localization of the *transducer of regulated cAMP response element-binding protein (CREB) 2* (*TORC2*) gene. (**A**) The mRNA expression level of the TORC2 gene in different tissues. *Glyceraldehyde 3-phosphate dehydrogenase* (*GAPDH*) was used as the housing gene (**B**,**C**) Real time qPCR was used for the detection of the TORC2 gene in preadipocytes transfected with OE-NC, OE-TORC2, siNC, and siTORC2. (**D**) Western blot analysis of the TORC2 protein gene in adipocytes transfected with OE-NC, OE-TORC2, siNC, and siTORC2. The analysis in (**D**) shows the overexpression efficiency of OE-TORC2 compared with OE-NC, and the silencing efficiency of TORC2 after transfecting the siTORC2 compared with siNC. (**E**) Subcellular localization of TORC2, immunofluorescence with the anti-TORC2 antibody (red), and nuclei were visualized with DAPI (blue) in bovine adipocytes. The right panel shows merged views. Pictures were captured though an Olympus IX71 microscope (OLYMPUS, Tokyo, Japan). One-way ANOVA and t-test were used for statistical analysis. Asterisks indicate significant variations. **** p < 0.0012.2. TORC2 promotes preadipocyte proliferation.

To elucidate the function of the bovine *TORC2* gene, the relative expression level of *TORC2* was identified in eight different tissues of Qinchuan cattle (Figure 1A). The expression level of the *TORC2* was highest in the abomasum followed by the liver, the small intestine, and the large intestine. The expression level of the *TORC2* was lower in the reticulum and rumen. Intramuscular adipose tissue expressed a higher concentration of *TORC2* mRNA as compared to the adrenal fat. Furthermore, to evaluate the role of the *TORC2* gene in bovine adipocytes, the adipocyte cells were transiently transfected with pcDNA3.1 blank (OE-NC), pcDNA3.1-TORC2 (OE-TORC2), siTORC2, and siNC. First, the transfection efficiency was confirmed through measurement of *TORC2* expression in different cells transfected with OE-NC, OE-TORC2, siNC, and siTORC2 (Figure 1A–C). The expression level of the *TORC2* gene in cells transfected with OE-TORC2 was significantly increased (*p* < 0.01) as compared to OE-NC (Figure 1A–C). Conversely, the expression level of *TORC2* in cells transfected with siTORC2 was significantly decreased (*p* < 0.01) as compared to siNC (Figure 1B,C). These findings suggested that the transfection experiment conducted in the present study was successful, and ensured the reliability of data in subsequent experiments. Moreover, immunofluorescence exhibited subcellular localization of the *TORC2* gene both in the cytosol and nucleus of the bovine preadipocytes (Figure 1E).

**Figure 2:**
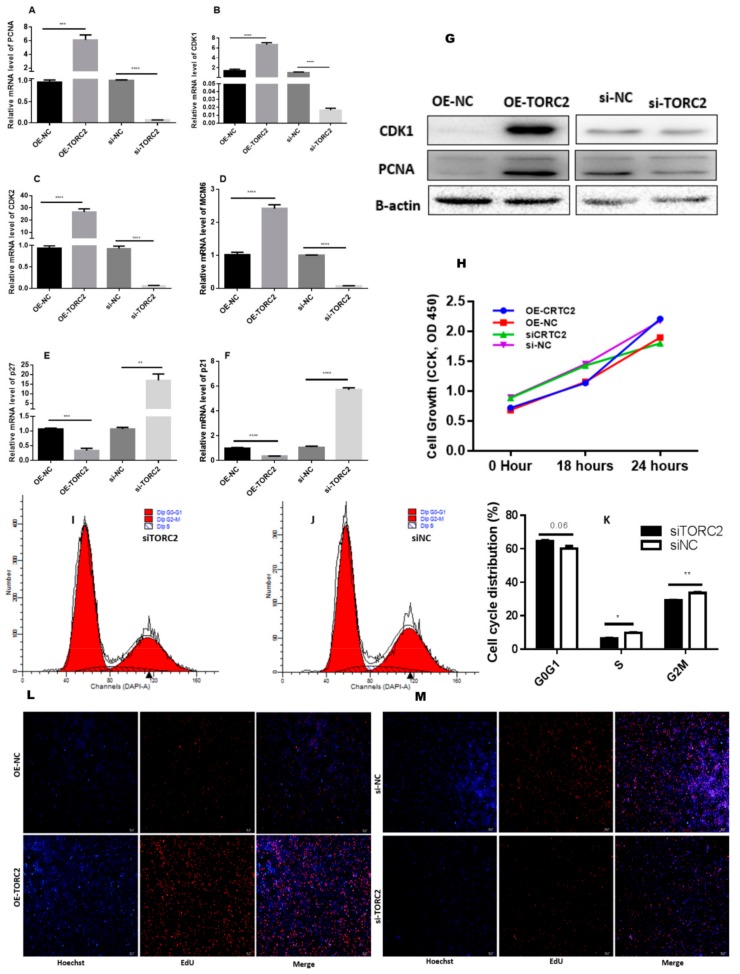
*TORC2* promotes adipocyte proliferation. *OE-NC*, *OE-TORC2*, *siNC,* or *siTORC2* were transiently transfected into bovine preadipocytes at 50% to 60% confluence, and cells were harvested at 24 h after transfection. (**A**–**F**) Real time qPCR was used for the detection of cell cycle genes, PCNA, *CDK1*, *CDK2*, *MCM6*, p21 and p27 24 h after transfection. (**G**) Western blot analysis of the cell cycle genes. (**H**) Cell count was measured using a cell count kit 8 (CCK-8), and the results show absorbance values at a wavelength of 490 after incubation with 10% CCK-8 solution for 3 h. The plots of cell cycle analysis in different cell cycle phases were compared (**I**–**K**) Cell cycle analysis was performed through a flow cytometer 24 h after transfection. (**L**,**M**) EdU (5-ethynyl-2′-deoxyuridine) assay was performed 24 h after transfection. Cells during DNA replication were stained by EdU (red), and the cell nuclei were stained with Hoechst (blue). The values represent mean ± SEM (*n* = 3). * *p* < 0.05 and *** *p* < 0.01.

Overexpression of *TORC2* significantly (*p* < 0.01) enriched cell cycle-related gene (*PCNA*, *CDK1, CDK2,* and *MCM6*) expression, both at the mRNA and protein level (Figure 2A–D,G), while the expression level of p21 and p27 was significantly suppressed (*p* < 0.01) (Figure 2E,F). Furthermore, a cell cycle assay performed through flow cytometry exhibited that a down-regulation of *TORC2* reduced the percentage of S-phase cells markedly (Figure 2I–K). Next, EdU (5-ethynyl-20-deoxyuridine) staining was used to investigate the role of *TORC2* in the proliferation of bovine pre- adipocytes. As shown in Figure 2L,M, over-expression of *TORC2* significantly increased the number of EdU labelled cells. However, a down-regulation of *TORC2* significantly decreased the ratio of EdU-labelled cells. Further, the CCk-8 assay results showed increased proliferation vitality of adipocytes transfected with *OE-TORC2* than *OE-NC*. In contrast, the cells transfected with *siTORC2* had lower proliferation vitality than those transfected with *siNC* (Figure 2H). Collectively, these findings elucidated that *TORC2* promotes adipocyte proliferation.

### 2.2. TORC2 Enhance Adipocyte Differentiation

**Figure 3:**
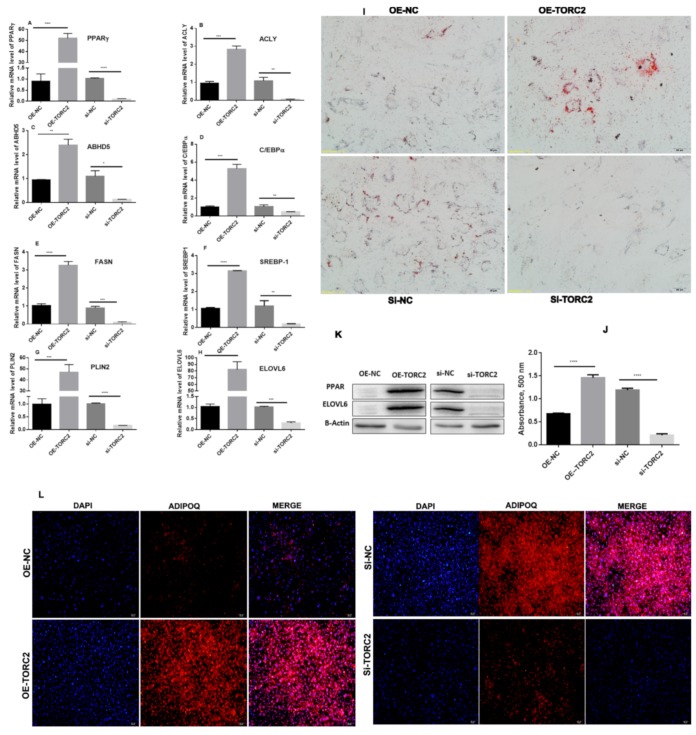
*TORC2* promotes bovine adipocyte differentiation. (**A**–**H**) Expression level of genes related to adipocyte differentiation. Relative levels of mRNA of *PPARγ*, *ACLY*, *ABHD5*, *CEBPα*, *FASN*, *SREBP-1*, *PLIN2*, and *ELOVL6* were measured by qRT-PCR. (**K**) The protein levels of PPARγ, ELOVL6 with reference protein β-ACTIN was measured through Western blotting. (**I**, **J**) Oil red O staining of bovine adipocyte cells at day 9 of differentiation transfected with *OE-NC, OE-TORC2, siNC,* or *siTORC2.* (**L**) Immunofluorescence of Adiponectin was performed at day 9 of differentiation of cells transfected with *OE-NC, OE-TORC2, siNC,* or *siTORC2.* The values represent the mean ± SEM (*n* = 3). * *p* < 0.05; ** *p* < 0.01; and *** *p* < 0.001.

To elucidate the role of the *TORC2* in bovine adipocyte differentiation, we first performed an Oil red O staining analysis (Figure 3I,J) and, as expected, overexpression of *TORC2* gene markedly decreased the lipid droplets in bovine adipocytes at day 9 of differentiation. In accordance with these results, the immunofluorescence of adiponectin protein in bovine adipocytes were markedly increased as well in the *OE-TORC2* and *siNC* groups and decreased in the *OE-NC* and *siTORC2* groups (Figure 3L). To further confirm the function of *TORC2* in the differentiation of bovine adipocytes, the expression levels of some adipogenic marker genes and regulators were detected. As shown in Figure 3A–H, the expression levels of the *PPARγ*, *ACLY*, *ABHD5*, *CEBPα*, *FASN*, *SREBP-1*, *PLIN2*, and *ELOVL6* genes were significantly enriched by *TORC2* (*p* < 0.05 and *p* < 0.01). Subsequently, these findings suggest that *TORC2* is a positive regulator of bovine adipocyte differentiation.

### 2.3. Identification of Transcription Start Site (TSS) of the TORC2 Gene

**Figure 4:**
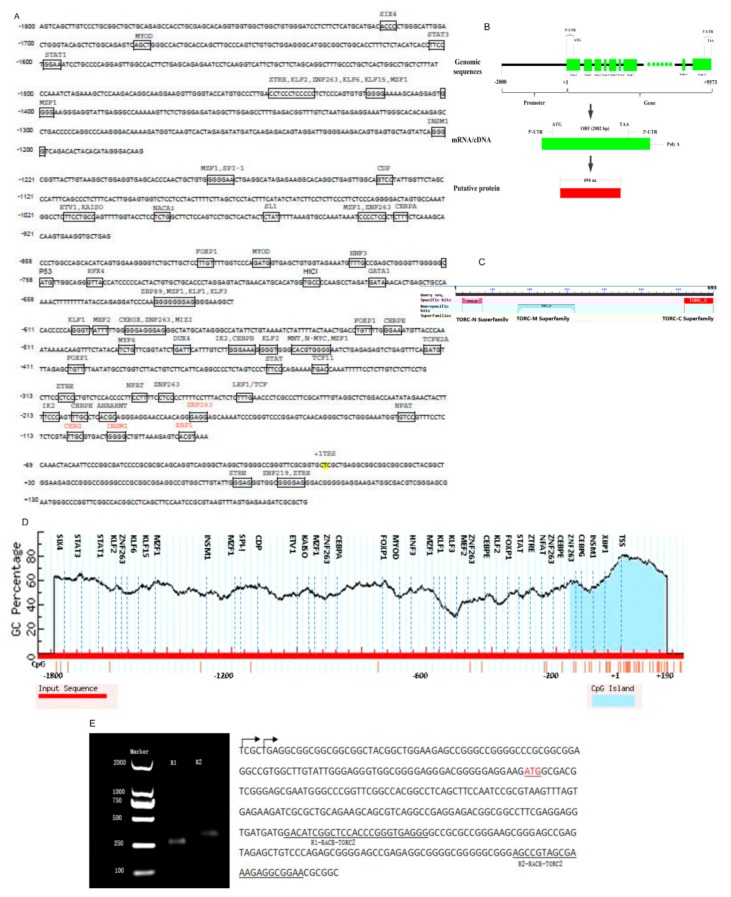
Structural characteristics of the bovine *TORC2* gene. (**A**) The 5′UTR promoter region sequence of the *TORC2* gene. The transcription start site has been highlighted with yellow color, transcription binding sites of the respective cis-acting elements in the promoter of *TORC2* gene have been boxed with their respective names shown above the line, while selected transcription factors (*C/EBPγ, XBP1, ZNF263,* and *INSM1*) were shown with a red color font in the core promoter region of *TORC2* gene. (**B**) The molecular structure of the *TORC2* gene with total length, number of exons, ORF, and number of amino acids. (**C**) The protein sequence has been shown with specific hits for domains site and super families in the protein sequence of the *TORC2* gene. Three domains hits, including TORC-N, TORC-M and TORC-C, have been shown in the protein sequence with their specific hit sites. (**D**) The CpG island is indicated with the blue color. Horizontal dash line indicates the GC percentage in the promoter region of *TORC*2 gene. The dash vertical lines show transcription factors binding sites with their respective names mentioned in the top. The horizontal red line indicates the input sequence of the *TORC2* gene promoter. The transcription start site is marked with +1. (**E**) Transcription start site of *TORC2* gene through RACE (for 5′ end). TORC2 5′ RACE PCR product gel electrophoresis band (left). The sequence of *TORC2* gene is (right), transcription start sites indicated with arrows, reverse primers sequence has been presented with underline sequence and translation start site (ATG) is shown with red color.

The 5′rapid amplification of cDNA ends (RACE) was performed to identify the *TORC2* gene transcription start site (Figure 4E). Two reverse primers (R1 and R2) produced two different bands of length 359 bp and 266 bp, respectively. Sequencing of the gene fragments explored two different positions of transcription start sites (TSS). The *RACE-R2 TORC2* primer revealed the same TSS as reported in the NCBI directory (NM_001076250.1), while the *RACE-R1-TORC2* primer revealed TSS tentatively to be located at 5 bp downstream of the NCBI reported TSS of the bovine *TORC2* gene.

### 2.4. Identification of Core Promoter Region of TORC2 Gene

**Figure 5:**
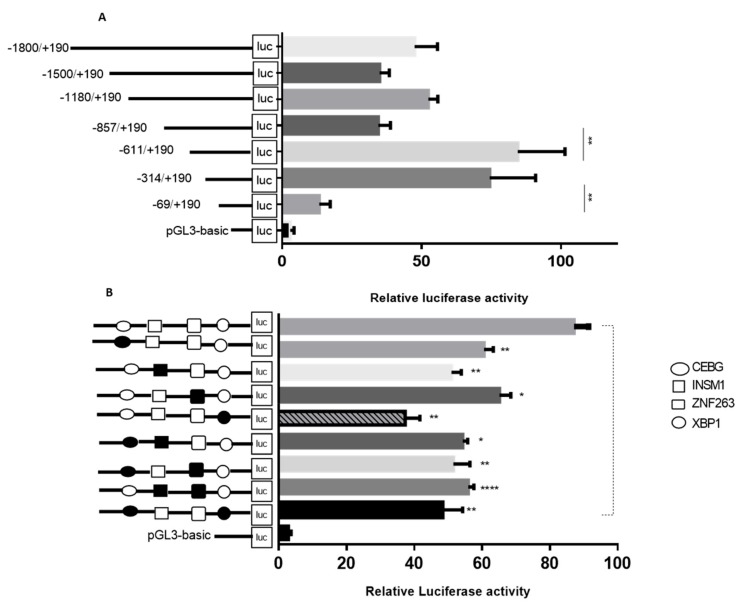
Luciferase reporter assay of the *TORC2* promoter and identification of the transcription start site. (**A**) Transcriptional activity of promoter fragments *TORC2-F1* (1990 −1800/+190), *TORC2-F2* (1690 −1500/+190), *TORC2-F3* (1370 −1180/+190), *TORC2-F4* (1047 −857/+190), *TORC2-F5* (801 −611/+190), *TORC2-F6* (504 −314/+190), *TORC2-F7* (259 −69/+190) and the pGL3-basic vector using dual-luciferase reporter assay. The reporter vector (pGL3-basic) was used as reference control to measure variation in the transcriptional activity in different fragment constructs through Tukey’s multiple comparison test. (**B**) Luciferase reporter assay conducted after site-directed mutation in the transcription factors binding sites of selected transcription factors (*C/EBPγ, XBP1, ZNF263* and *INSM1*) located in the core promoter region of the *TORC2* gene. Transcriptional activity of the two groups were compared through t-test with the activity of *TORC2-F6* (504 −314/+190).

To identify the core promoter region of the *TORC2* gene, seven serial reporter constructs were generated in the luciferase reporter vector through unidirectional deletions from the 5′ end. Transcriptional activities of the respective fragments were assessed in the bovine preadipocytes through Dual Luciferase Reporter Assay (Figure 5A, Table S2). The mean difference between the transcription activity of the *TORC2-F1* (1990 −1800/+190) and the pGL3-basic was (44.6) (*p* < 0.001). When the sequence was deleted from −1800 to −1500 bp the mean difference between the pGL3 basic and the *TORC2-F2* (1690 −1500/+190) reduced to 32.07 (*p* < 0.05). However, with further deletion from −1500 to −1180 bp, the transcriptional activity of the *TORC2-F3* (1370 −1180/+190) construct increased significantly (*p* < 0.001). Further deletion from -1180 to 857 bp caused a reduction in the transcriptional activity with the mean difference of (31.65) (*p* < 0.05). However, the highest transcriptional activity was found in the cells transfected with *TORC2-F5* (801 −611/+190) with a mean difference value of (81.60) (*p* < 0.001) as compared to the pGL3-basic vector. Further unidirectional deletion in the 5′ end sequence of the *TORC2-F5* (801 −611/+190) fragments to *TORC2-F6* (504 −314/+190) caused a reduction in the transcriptional activity with significant (*p* < 0.05) mean difference (71.54). When deletion was extended to −69 bp from −314 bp in *TORC2-F7* (259 −69/+190), the transcriptional activity and mean difference (10.45) reduced significantly (*p* < 0.001). These results indicated that the functional minimal promoter of the *TORC2* gene is located in the position −314 to −69 upstream of the transcription start site.

### 2.5. Roles of C/EBPγ, XBP1, INSM1 and ZNF263 in Transcriptional Regulation of TORC2 Gene

Bioinformatics tool Genomatix Mathinspector software (Intrexon Bioinformatics Germany GmbH available online: http://www.genomatix.de/index.html accessed on 3 September 2019) was used for the identification of the transcription factor (TF) binding sites in the core promoter region of the bovine *TORC2* gene with the cut off value of 90%. Various TF binding sites were found as shown in Figure 4A,D. Based on the in silico predicted location within the core promoter region—especially within the CpG island—proximal placement to transcription start site, and previously reported studies, four important transcription factors *C/EBPγ, XBP1, INSM1* and *ZNF263* were selected for further confirmation. The in silico analysis further revealed the absence of consensus TATA and CCAAT boxes in vicinity of the transcription start site (TSS) (Figure 4A). However, MethPrimer computer online suit (The Li lab Home page available online: http://www.urogene.org/methprimer/ accessed on 3 September 2019) explored one CpG island located from (−159 to +134 bp) relative to the TSS in the *TORC2* promoter region (Figure 4D). The selected transcription factor binding sites were located within the CpG Island.

For validating the regulatory roles of the selected transcription factors, mutated fragment plasmids were constructed. Bovine preadipocytes cells were transiently transfected with the mutated and non-mutated plasmids of the respective TF binding sites. The dual luciferase reporter assay explored a significant variation in the transcriptional activities of different mutated transcription factor binding sites (Figure 5B). Transcription activities of the mutated constructs of *C/EBPγ*, *XBP1*, *INSM1* and *ZNF263* were significantly reduced (*p* < 0.01 and *p* < 0.05). To further validate the roles of these TF binding sites in the core promoter region of the *TORC2* gene, double TF binding sites mutation plasmid were constructed. Figure 5B shows a significant decrease (*p* < 0.01 and *p* < 0.05) in the transcription activities of all the double mutated plasmids compared with the non-mutated plasmid fragment *TORC2-F6* (504 −314/+190). These results suggest that the transcription factors *C/EBPγ*, *INSM1*, *XBP1* and *ZNF263* are positive activators of the *TORC2* gene.

### 2.6. Genetic Interaction with Transcription Factors

**Figure 6:**
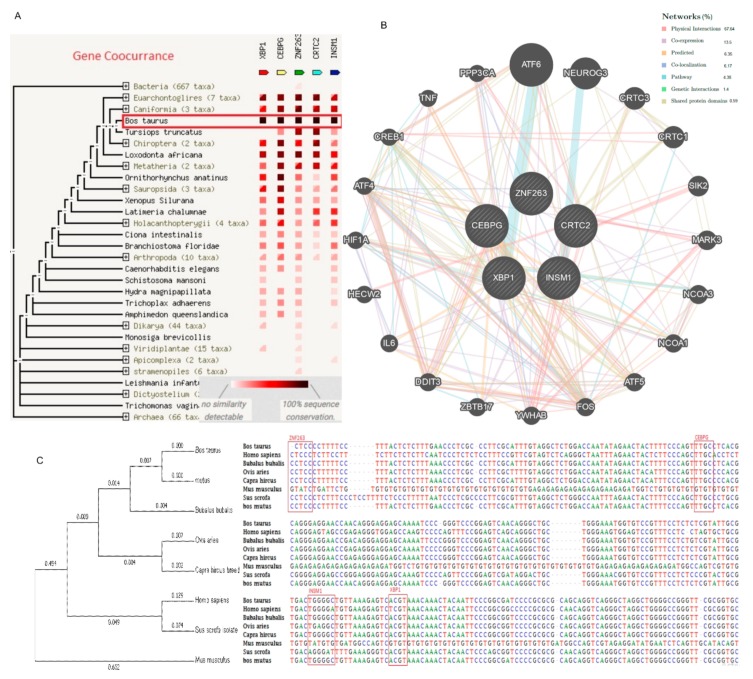
Genetic interaction and detailed phylogenetic tree with sequence alignment of the *TORC2* gene and selected transcription factors within the promoter region of different animals. (**A**) Gene co-occurrence of *TORC2* gene with *C/EBPγ*, *XBP1*, *INSM1* and *ZNF263* transcription factors in *Bos taurus*. The color denotes—for each gene of interest—the similarity of its best hit in a given STRING genome. Similarities in these presence/absence profiles can predict interactions among target genes. (**B**) Interaction among the target genes (shown in the center). (**C**) Phylogenetic tree and multiple sequence alignment analysis of *TORC2* promoter region (−300 bp) for *ZNF263*, *C/EBPγ*, *INSM1* and *XBP1* transcription factor binding sites in cattle, human, water buffalo, sheep, goat, house mice, pig and wild yak. Selected transcription factor binding site sequences were found conserved in cattle, human, water buffalo, sheep, goat, pig and wild yak. However, variation was found in the DNA sequence of house mice as compared with other species.

Online bioinformatics tools GeneMANIA (University of Toronto Home Page available: https://genemania.org/ on 3 September 2019) and STRING database (Swiss Institute of Bioinformatics available online: https://string-db.org/ accessed on 3 September 2019) were applied for the identification of genetic interaction among the *TORC2* gene and its four transcription factors. The in silico analysis showed a close interaction among these genes. Genetic interaction among the target genes were denoted by colors (Figure 6A), the black color represents the highest interaction between the target genes in different species. In *Bos taurus,* there is a high interaction among the *TORC2, ZNF263, INSM1, C/EBPγ* and *XBP1* as shown through the concurrence of the genes. The predicted network interaction among the *TORC2* and the selected transcription factors (*C/EBPγ*, *XBP1*, *ZNF263*, and *INSM1*) revealed a 67.64% physical interaction. The co-expression, co-localization and shared protein domains structures were 13.50%, 6.17%, and 0.59%, respectively.

### 2.7. Silencing of C/EBPγ, ZNF263, XBP1 and INSM1 Transcription Factors

**Figure 7:**
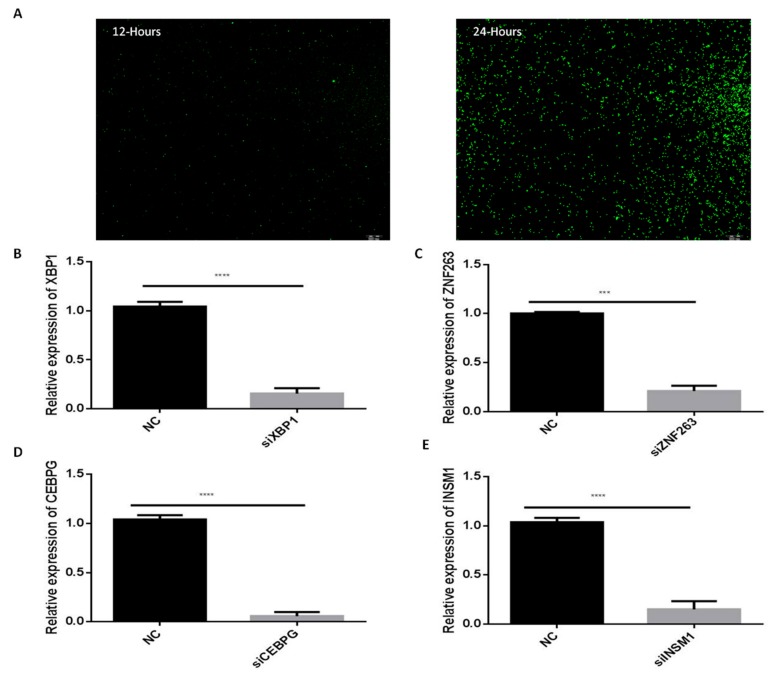
Transfection and interference efficiencies of siRNAs. (**A**) FAM-labeled siRNA (NC). FAM-Labeled siRNA-NC was transfected into bovine adipocytes and transfection was checked after 6, 12 and 24 h intervals. Pictures were captured though an Olympus IX71 microscope (OLYMPUS). (**B**,**E**) the transcription factors *XBP1*, *ZNF263*, *C/BEPγ*, and *INSM1* were down-regulated through small interference RNA (siRNA). The β-actin was as a housekeeping gene. Relative mRNA expression levels were normalized with NC. The values represent mean ± SEM (*n* = 3). * *p* < 0.05; *** *p* < 0.01; and **** *p* < 0.001.

The selected transcription factors *C/EBPγ, ZNF263, XBP1* and *INSM1* were silenced through siRNAs. First, the transfection efficiency of the siRNA was detected through FAM-labeled negative control siRNA (gene pharma, Shanghai, China). The transfection efficiency was checked at 12- and 24-h intervals (Figure 7A). The highest transfection efficiency was found after 24 h of transfection. Upon completion, the interference efficiency of the siRNAs against the selected transcription factors was evaluated. These siRNAs significantly reduced (*p* < 0.01) the mRNA expression levels of the *XBP1*, *ZNF263*, *C/EBPγ* and *INSM1* as compared to the negative control (NC) (Figure 7B–E). These findings showed that the transcription and interference efficiencies of these siRNAs were successful.

**Figure 8:**
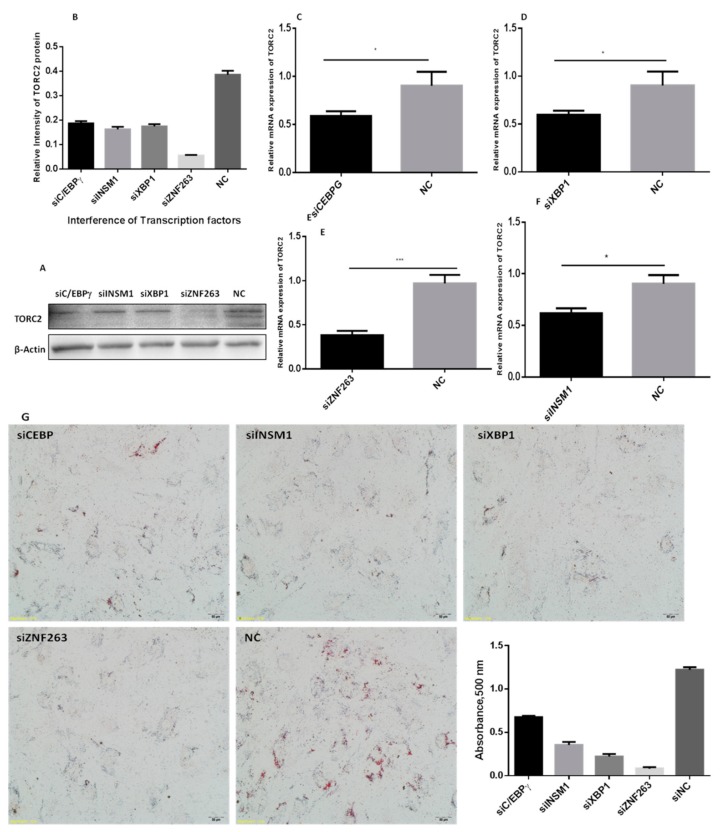
Expression level of the *TORC2* gene and cell phenotypes of adipocytes transfected with siRNAs of selected transcription factors. (**A**,**B**) Protein level of TORC2 in preadipocytes transfected with siC/EBPγ, siXBP1, siZNF263, siINSM1 and NC. (**C**–**F**) Shows mRNA expression level of *TORC2* gene in preadipocytes transfected with siC/EBPγ, siXBP1, siZNF263, siINSM1 and NC. *GAPDH* was used as the housekeeping gene. The values represent mean ± SEM (*n* = 3). * *p* < 0.05 and *** *p* < 0.01. (**G**) Shows the cell phenotypes of adipocytes transfected with siC/EBPγ, siXBP1, siZNF263, siINSM1 and NC stained with Oil red O staining.

The interference of the selected transcription factors significantly altered the expression of the *TORC2* gene both at the mRNA and protein levels (Figure 8A–F). The expression of the *TORC2* gene mRNA and the protein levels significantly reduced (*p* < 0.05) with the down regulation of the *C/EBPγ*, *XBP1*, *INSM1* and *ZNF263* transcription factors as compared to the NC-group.

### 2.8. Oil Red O Staining

Results of the Oil red O staining showed a distinct variation in the number and diameter of lipids droplets in the adipocytes transfected with the siRNAs against the transcription factors *C/EBPγ*, *XBP1*, *INSM1, ZNF263* and NC (Figure 8G). The lipid droplets were substantially decreased both in number and size in cells transfected with transcription factors *C/EBPγ, XBP1, INSM1* and *ZNF263* siRNAs.

### 2.9. DNA-Protein Interaction through EMSAs

**Figure 9:**
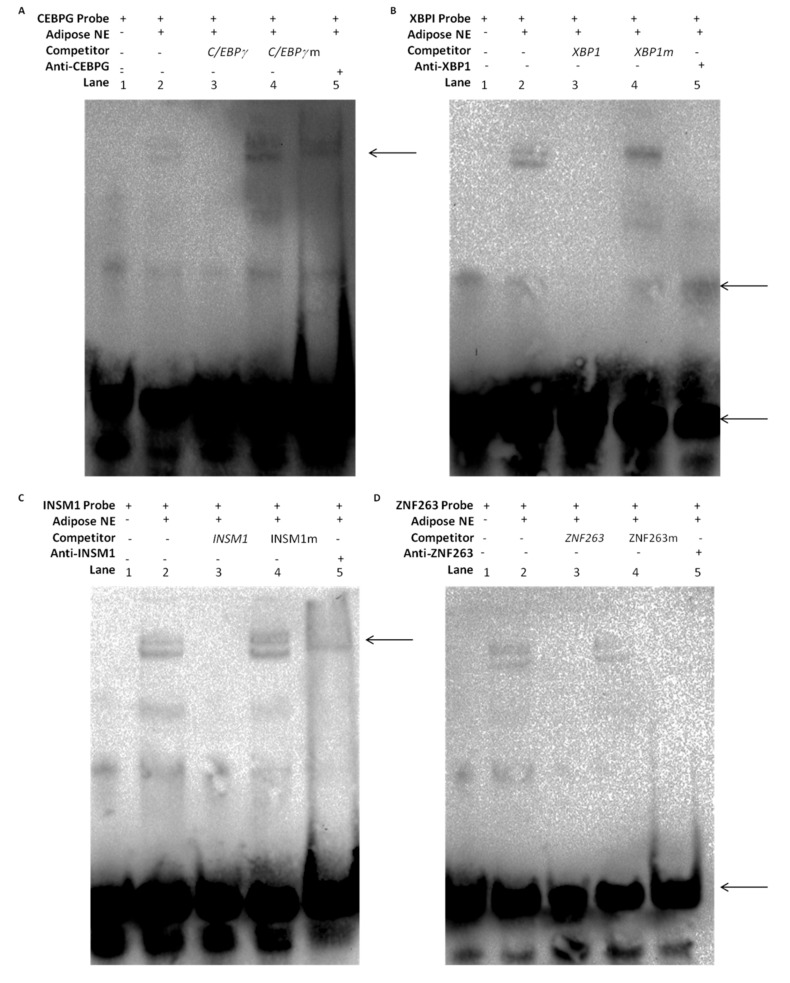
Electrophoretic mobility shift assay (EMSA) shows in vitro DNA-protein interaction of *C/EBPγ*, *XBP1*, *INSM1* and *ZNF263* transcription factors to the *TORC2* promoter. The nuclear protein extracts of bovine preadipocytes were incubated with the *C/EBPγ* (**A**), *XBP1* (**B**), *INSM1* and *ZNF263* (**D**) free probes (lane 2), 10× non biotinated probes (lane 3), 10× mutated probes (lane 4) and the super shift migrated of DNA-Protein with anti-CEBPG, Anti-XBP1, Anti-INSM1 and Anti-ZNF263 antibodies complexes (lane 5) (**A**–**D**). (**A**–**C**) the arrow indicates the supershift of the protein-DNA complexes, (**B**,**D**) and the bottom arrows indicate that the amount of the main complexes was clearly decreased.

An electrophoretic mobility shift assay was performed to confirm the roles of *C/EBPγ, XBP1, INSM1* and *ZNF263* transcription factors in the regulation of the *TORC*2 gene. It was revealed that there was an interaction of the DNA of the transcription factor with the nuclear protein extracted from the bovine preadipocytes. As shown in Figure 9, the bovine preadipocytes nuclear protein was bound with the 5′-biotin labeled probes of the *C/EBPγ*, *XBP1*, *INSM1* and *ZNF263* that formed DNA-protein complexes (lane-2, Figure 9A–D). The nuclear proteins were then incubated with 10X competition probes (specific, non-biotinated probes) and the protein-DNA complex disappeared (lane-3, Figure 9A–D). However, the protein-DNA complex did not change when mutated probes were added (lane-4, Figure 9A–D). Interestingly, the supershift of the protein-DNA complexes with 10 µg of anti-C/EBPγ, anti-XBP1, anti-INSM1 and anti-ZNF263 antibodies migrated upward (lane-5 Figure 9A–C). Although these experiments did not reveal a specific supershifted product at the binding sites of the *XBP1* and *ZNF263*, the amount of the main complexes was however clearly decreased (lane-5, Figure 9B,D). One possible explanation could be that the super-shifted product may be of high molecular weight polymer and could be stuck in the top of the well, which caused a reduction in the gel mobility shift (lane-5 Figure 9D). These results confirmed that *C/EBPγ, XBP1, INSM1* and *ZNF263* transcription factors can specifically bind with the promoter sequence of *TORC2* gene.

## 3. Discussion

Fat deposition, especially intramuscular fat, is considered important in beef production as an indicator of beef-eating quality [31,32]. The Qinchuan cattle are an indigenous Chinese breed, characterized by a dominant *Bos taurus* ancestry [33,34]. The breed exhibits good growth rates in Chinese production systems; however, the carcass quality traits—especially intramuscular fat content—are lower than those of exotic cattle breeds such as the Wagyu [35,36,37]. Low intramuscular fat is a significant industry issue and its effects on meat quality, and consumer satisfaction is well documented. Therefore, to improve the carcass quality of the Qinchuan breed it is appropriate to explore the molecular mechanism of bovine adipogenesis in Qinchuan cattle.

Adipogenesis is a complex and precisely orchestrated process, characterized by an increased number of adipocytes and increased lipid storage in adipocytes. Adipocytes are derived from the existing pool of preadipocytes, which are differentiated in response to the proper signal [38,39]. Therefore, it is very important to elucidate the molecular mechanism underlying adipogenesis. The *TORC2* is a well-known regulator of adipogenesis [13,40,41]. In the present study, a high expression of *TORC2* in abomasum and small intestine is in line with the findings of Liuqin et al. [42]. They concluded that the AMP-activated protein kinase (AMPK) pathway, which is regulated by the *TORC2* gene, is mainly responsible for water and ionic homeostasis in the small intestine in pigs [42]. Moreover, high expression in the liver is in line with findings of previous studies [21,43,44], where its core function is glucagon-mediated activation of hepatic gluconeogenesis [18,20,21] to maintain energy balance in vital tissue of the body [45,46]. Additionally, we found a high expression level of the *TORC2* gene in the intramuscular adipose tissue as compared to adrenal fat. The protein was localized in both the nucleus and the cytosol of bovine—where it performs its functions differently. Under basal conditions, the TORCs are sequestrated in the cytosol through phosphorylation-dependent fashion with dimeric 14-3-3 proteins. The cAMP and calcium pathways activate its release from 14-3-3 proteins, selectively activating the TORC phosphatase calcineurin and inhibiting TORC kinases such as the salt-inducible kinases (SIKs) [9,47,48]. However, upon dephosphorylation, the TORCs translocate into the nucleus to co-activate CREB transcription and to regulate downstream target genes [49,50,51,52]. The intracellular localization of TORCs is controlled by phosphorylation and dephosphorylation through the cAMP and calcium signaling pathways [9,53]. This translocation of *TORC2* is an essential and conserved step in the activation of cAMP-responsive genes [54]. These findings indicate that *TORC2* may regulate adipogenesis. Moreover, in the present study, the role of *TORC2* in bovine adipocyte proliferation was confirmed through cell cycle staining flow cytometry, cell counting assay, 5-ethynyl-2′-deoxyuridine staining (EdU), and mRNA and protein expression analysis of proliferation-related marker genes. In addition, Oil red O staining analysis, immunofluorescence of adiponectin, mRNA and protein level expression of lipid related marker genes confirmed the role of *TORC2* gene in the positive regulation of bovine adipocyte differentiation. The present study has identified the *TORC2* gene as a potential positive regulator of adipogenesis. These findings imply that *TORC2* is a novel hallmark for dealing with the problem of low intramuscular fat in cattle breeding program.

After elucidating the function of *TORC2* gene in bovine adipogenesis, an attempt was made to explore the transcriptional regulation of the *TORC2* gene in preadipocytes. Transcriptional regulation is a crucial biochemical regulatory mechanism in all living organism. It is organized by the orchestration of regulatory proteins and transcription factors. Therefore, the RNA level can be tuned precisely through various mechanisms, such as the copy number of transcribed RNA and spatiotemporal regulation of gene transcription. Transcriptional regulation allows the cell or organism to respond to various extracellular and intracellular signals and adopt accordingly. The promoter region is a DNA fragment, which may bind transcription factors, RNA polymerases, and other proteins for the transcription initiation of the particular gene [55]. Generally, the promoter is located proximal to the transcription start site (TSS) of a gene and upstream towards the 5′ region of the sense strand [56]. Therefore, in the present study, we first identified the TSS, core promoter region and then four important transcription factors *C/EBPγ*, *XBP1*, *ZNF263* and *INSM1* genes were selected to investigate their roles in the regulation of the bovine *TORC2* gene. These TFs were located in the core promoter region (−314 to +190) relative to the TSS. One CpG island was found in the promoter region of *TORC2* gene. However, neither a TATA nor CCAATT box was identified in the proximal promoter region. These observations are consistent with the findings where the TATA box is present only in 10% to 20% of the eukaryotic promoter sequence and is not a dispensable transcription factor. [57,58,59]. Previous literature supports the role of the *TORC2* gene in DNA methylation through regulation of DNA mismatch repair system [60]. DNA methylation is a regulatory process for the normal gene expression and function [61,62,63]. DNA mismatch repair is one of the tools of DNA methylation for sustaining DNA stability and gene expression [64]. In the present study, analysis of the promoter sequence from −1800 to +190 bp of the *TORC2* gene explored the region between −314 to +190 bp as the core promoter region, which contained the consensus transcription factor motifs for the *C/EBPγ*, *XBP1, ZNF263* and *INSM1*. Bioinformatics analysis showed location of these TFs within the GC-rich CpG island of *TORC2* gene—which suggests that the function of the *TORC2* is under epigenetic regulation.

The transcription factors CCAAT/enhancer binding proteins (C/EBPs) belong to the leucine zipper transcription factors family, which plays a vital function in cellular proliferation and differentiation. The *C/EBPγ* is a member of this family, which is expressed ubiquitously in tissues and has the binding affinity with regulatory motifs of immunoglobulin long chain enhancer and promoter. The *C/EBPγ* modulates the C/EBPs activity in a cell-specific manner [65,66]. The interaction of the *CEBPγ* with the *CEBPβ* causes cell proliferation and inhibition of cell senescence [67]. The *XBP1* transcription factor is a major regulator of endoplasmic reticulum (ER) and unfolded protein response (UPR) or stress response, activated by certain stressors including protein and fat overloading, which causes increase endoplasmic reticulum capacity. *XBP1* regulate terminal stage differentiation of preadipocytes to adipocytes, and consequently elevate the secretion of the unfolded protein response (UPR) as a stress response in the endoplasmic reticulum to increase the rate of protein and lipid biosynthesis [68]. In the human model, *XBP1* is main regulator of cell growth and survival; the expression of *XBP1* is directly proportional to up-regulation of P13/mTOR-TORC2 signaling pathway, which is vital for cell proliferation and growth [69]. *INSM1* regulates the chromatin modifying factors of histone deacetylases; histone modification performs vital roles in various biological processes and the expression and regulation of genes [70]. *INSM1* is a zinc finger transcription factor, which performs a vital role in the regulation of target genes or indirectly through the stimulation of growth factor signaling pathways [71]. *INSM1* with the interaction of the RACK1 gene promotes P13/AKT signaling pathway as well [72]. The *ZNF263* is a member of the Zinc finger motifs, which is a C2H2 protein that contains nine zinc finger domains, one SCAN domain and one KRAB repression domain. Genome-wide ChIP sequencing identified more than 5000 binding sites for this transcription factor in the promotion of multiple genes that regulate their target genes either as a repressor or activators [73,74]. In the present study, the luciferase reporter assay of the site directed mutation in the core sequence of *C/EBPγ, XBP1, INSM1* and *ZNF263* significantly reduced the transcriptional activity of the *TORC2* gene. Moreover, interference through siRNA caused a significant (*p* < 0.05) reduction in the expression of the *TORC2* gene both at the mRNA and protein levels. Lipid droplets quantity and diameter was decreased in adipocytes transfected with siRNAs against the selected TFs as well, as compared with negative control group. Furthermore, EMSA assay additionally confirmed the binding of *C/EBPγ*, *XBP1, INSM1* and *ZNF263* in the nuclear extract of adipocytes in the promoter region of the *TORC2* gene. From these findings, we can conclude that *C/EBPγ*, *XBP1, INSM1* and *ZNF263* regulate *TORC2* gene as activators in the core promoter of *TORC2* gene. Although we identified the role of *C/EBPγ*, *XBP1, INSM1* and *ZNF263* in the regulation of *TORC2* gene further study is however needed to investigate the roles of these TFs in adipogenesis.

## 4. Materials and Methods

### 4.1. Ethics Statement

The procedures for animal handling for experiments were approved by EAMC (Committee of experimental animal management) at Northwest Agriculture and Forestry University, China. Moreover, all applicable rules and regulation of the organization and government were followed regarding the ethical use of experimental animals.

### 4.2. Tissue Collection and mRNA Expression

For tissue collection, three mature Qinchuan cattle (24 months old) were selected from the National Beef Cattle Improvement Research Centre. The animals were dressed in a local abattoir under standard procedure of animal stunning, exsanguinations, and skinning. To measure the relative expression of *TORC2* gene, eight tissues including rumen, reticulum, abomasum, small intestine, large intestine, liver, muscular fat and adrenal fat were aseptically collected in liquid nitrogen. The total RNA was extracted from the tissue using TRIzol™ Reagent (Invitrogen, Thermo Fisher Scientific, Inc. Waltham, MA, USA). The integrity (quantity and quality) of the extracted total RNA was checked through an optical density of 260 and ratio of the optical density (OD) of 260/280 using the Nano Quant plate TM (Infinite M200 PRO, TECAN, Switzerland), and was further verified through 1% agarose gel. cDNA libraries were constructed using PrimeScriptTM RT reagent kit with gDNA eraser (Perfect Real Time, Takara, Beijing, China). Quantitative real time (RT-PCR) was performed following the manufacturer’s protocol of Sybr Premix EX Taq Kit (Takara, Dalian, China) using thermocycler 7500 system SDS V 1.4.0 (Applied Biosystem, Foster, CA, USA). Bovine *β-Actin* and *GAPDH* (glyceraldehyde-3-phosphate dehydrogenase) genes were used as an endogenous control. The thermocycling conditions were: pre heating at 95 °C for 5 min, a total 34 cycles of denaturation at 95 °C for 30 s, annealing temperature at 60 °C for 30 s and extension temperature at 72 °C for 30 s. The relative mRNA expression levels were calculated using 2^−ΔΔCt^ method [75].

### 4.3. Isolation of Bovine Primary Preadipocytes

Bovine preadipocyte cells were collected from a healthy newborn calf (5 days old) of the Qinchuan cattle breed using the methods previously described [76,77,78]. The back-fat tissue from the longissimus dorsi muscle area was extracted under aseptic conditions. The muscle was first washed with 75% ethanol and then dissected using sterile and sharp surgical-curved scissors. The sample was washed with 1XPBS supplemented with 10% penicillin/streptomycin antibiotics three times and immediately transferred to the cell culture room. The adipose tissues were dissected from the blood vessels and connective tissues under a stereo dissecting microscope with the help of sterile forceps. These tissues were then minced and subjected to enzyme digestion with collagenase I, 0.25% (Sigma, Shanghai, China) for 1 h at 37 °C in a shaking water bath. This digestive mixture was neutralized with equal volume of 10% FBS (Invitrogen, Waltham, MA, USA), the mixture was strained using 100 μm and then 40 μm strainers. The filtrate was centrifuged at 1500× *g* for 10 min, and the cell pellet was washed twice with DMEM-F/12 medium (Gibco, Grand Island, NY, USA) without serum. The cell pellet was then resuspended with DMEM-F/12 medium supplemented with 10% FBS and seeded in 60 mm collagen coated cell culture plates and incubated at 37 °C and 5% CO_2_ for 1 h. One hour later, the medium was changed, and washed three times with 1XPBS to remove the debris and free-floating cells.

### 4.4. Cell Culture and Immunofluorescence

Bovine preadipocyte cells were cultured in a 24-well culture plates. The cultured cells were fixed with 4% paraformaldehyde for 15 min. The fixed cells were washed with phosphate buffer saline (1× PBS) which contains 0.0067M (PO_4_), calcium and magnesium, with pH 7.0 to 7.2 (HyClone^TM^, Logan, UT, USA). After washing with 1× PBS the cells were permeabilized with 0.2% Triton X-100 for 15 min. For immunofluorescence, the cells were incubated with the primary anti-CRTC2 antibody (1:200 dilution, Invitrogen, MA5-15710) and anti-adiponectin antibody (1:300, Bioss, Beijing, China, Cat No bs-0471R) overnight at 4 ˚C. The cells were washed with 1XPBS and incubated with secondary anti-rabbit IgG H&L antibody (Alexa Fluor^®^ 555) (1:1000 dilution, Abcam, Cambridge, UK), and protected from light at 37 ˚C for 1 h. The nuclei were stained under dark with DAPI (Sigma-Aldrich, St. Louis, MO, USA) at room temperature for 15 min. DAPI was used at the final concentration of 1 μg/mL. Immunofluorescence images were taken with an Olympus IX71 microscope (OLYMPUS, Tokyo, Japan).

### 4.5. EdU Proliferation Assay

The 5-ethynyl-2′-deoxyuridine (EdU) assay was performed using a cell light EdU DNA proliferation kit (RiboBio, Suzhou, China). Bovine preadipocytes were seeded in 24-well cell culture plates and transfected at 50% to 60% density with OE-NC, OE-TORC2, siNC, or siTORC2. After 24 h, the cells were incubated with 5-ethynyl-2′-deoxyuridine medium for 2 h. After 2 h incubation, the EdU assay was performed according to the manufacturer’s protocol.

### 4.6. Cell Cycle Assay through Flow Cytometry

Bovine preadipocytes were seeded in six-well cell culture plates. The cells were transfected with OE-NC, OE-TORC2, siNC, or siTORC2. After 24 h, the cells were harvested, washed with 1XPBS, and re-suspended with 1× PBS containing 1 mL DNA staining solution and 10 μL permeabilization solution (Multisciences, Hangzhou, China). The suspension was vortexed for 15 s and incubated for 30 min in the dark at RT. The cell cycle was analyzed through flow cytometry (FACS CantoTM II, BD BioSciences, San Jose, CA, USA) by counting 20,000 cells.

### 4.7. CCK-8 Assay

Bovine preadipocytes were seeded in 96-well plates and transiently transfected with OE-NC, OE-TORC2, siNC, or siTORC2. The cell proliferation was detected after 0, 18 and 24 h of transfection using a TransDetect CCK kit (TransGen Biotech, Beijing, China), according to the manufacturer’s protocol. Cells were added 10 µL of CCK solution and to each well, and incubated for 3 h at 37 °C in a 5% CO_2_ cell incubator. The absorbance was measured using a Nano Quant plate TM (Infinite M200 PRO, TECAN, Switzerland) reader at a wavelength of 450 nm.

### 4.8. 5′-Rapid Amplification of cDNA Ends (RACE)

Transcription start site (TSS) of *TORC2* gene was identified through RACE- Kit (SMARTer, 5′) (Takara, Beijing, China) as per the manufacturer′s instructions (Clontech Inc., CA, USA). Total RNA was extracted using TRIzol™ Reagent (Takara, Beijing, China) from the muscular fat of Qinchuan cattle. RACE ready 5′-end first strand cDNA was synthesized through SMARTer RACE 5′ Kit (Takara, Beijing, China) PCR was performed for the amplification of RACE ready cDNA using two different gene specific reverse primers (Table S2) located between exon 1 and 2 of the *TORC2* gene and Universal Primer A Mix (Takara, Beijing, China) was used as forward primer. Nested PCR was conducted with the conditions as mentioned previously [79,80] using SeqAmp DNA polymerase PCR enzyme (Clontech Inc., CA, USA). Touchdown PCR was performed with the conditions of five cycles at 94 °C for 30 s, 72 °C for 3 min; then, five cycles at 94 °C for 30 s, 70 °C for 30 s, 72 °C for 3 min, and; 25 cycles at 94 °C for 30 s, 68 °C for 30 s and 72 °C for 3 min. The PCR product was isolated from the 1% agarose gel through gel extraction kit (Omega Bio-tek, Norcross, GA, USA) and cloned into pMD t-vector (simple) (Takara, Beijing, China). Selected clones were sequenced through Sangon (Shanghi, China).

### 4.9. DNA Extraction and Amplification of TORC2 Gene Promoter

The genomic DNA was isolated from the blood samples of Qinchuan cattle using the phenol chloroform method (Sambrook and Russell, 2001) and DNA extraction kit (Omega Bio-tek, Norcross, GA, USA). The extracted DNA was diluted to make the final concentration as 50 ng/μL and stored at −20 °C. Gene specific primers (TORC2-Fragment 1, Table S1) were used to amplify a 1990 bp promoter region, including TSS (transcription start site) of the bovine *TORC2* gene (NCBI accession No NC_037330.1 from 16477661 to 16487214). Qinchuan cattle genomic DNA was used as a template for the PCR amplification of *TORC2* gene promoter, using KOD plus neo enzyme (Toyobo, Tokyo, Japan). Thermocycling (PCR) was performed using three-step cycle conditions with pre-denaturation at 94.0 °C for 5 min followed by 34 cycles of denaturation at 97 °C for 30 s, annealing (Tm of the primers used, see Table S1) for 30 s and final extension at 72.0 °C for 45 s. The PCR product was cloned into pMD19 (simple) (Takara, USA).

### 4.10. Cloning of TORC2 Gene Promoter

**Figure 10:**
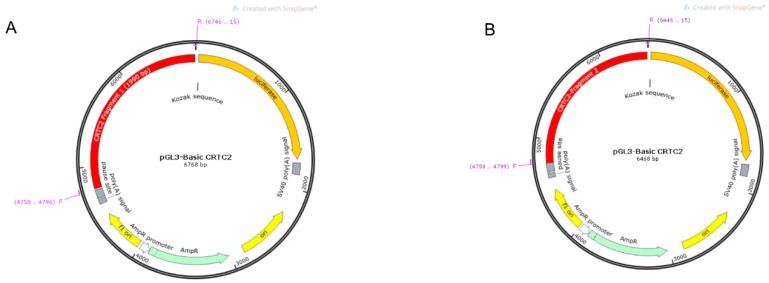

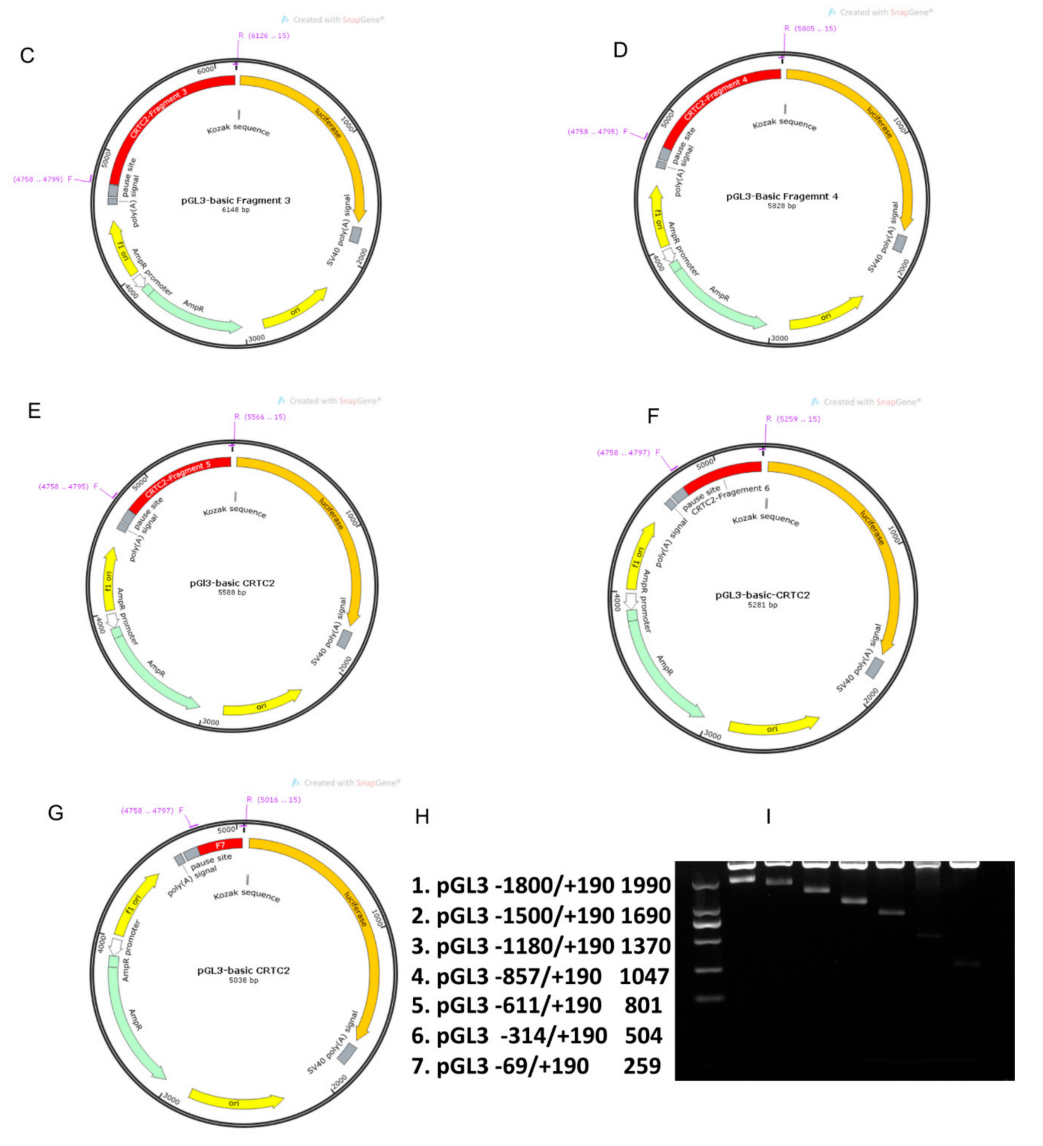
Amplification and cloning of *TORC2* gene into pGL3-basic vector. (**A**–**G**) Seven different fragments of TORC2 gene have been inserted into pGL3-basic vector and presented graphically through SnapGen software. (**H**,**I**) Indicates amplification of *TORC2* gene seven fragments ligated into pGL3-basic vector and after the enzyme cut the seven fragments, including TORC2-F1 (1990 −1800/+190), TORC2-F2 (1690 −1500/+190), TORC2-F3 (1370 −1180/+190), TORC2-F4 (1047 −857/+190), TORC2-F5 (801 −611/+190), TORC2-F6 (504−314/+190) and TORC2-F7 (259−69/+190).

The putative transcription factor binding sites were identified through online software Genomatix suit (Intrexon Bioinformatics Germany GmbH available online: http://www.genomatix.de/index.html accessed on 3 September 2019). The CpG Island in the prompter region of the *TORC2* gene was predicted through an online website Meth Primer (The Li lab Home page available online: http://www.urogene.org/methprimer/ accessed on 3 September 2019). Based upon the predicted transcription factor binding sites and position of the CpG Island, different fragment primers were designed. In total, seven different fragments were amplified through gene specific primers TORC2-F1 (1990 −1800/+190), TORC2-F2 (1690 −1500/+190), TORC2-F3 (1370 −1180/+190), TORC2-F4 (1047 −857/+190), TORC2-F5 (801 −611/+190), TORC2-F6 (504 −314/+190) and TORC2-F7 (259 −69/+190) with unidirectional deletion of the bovine *TORC2* gene promoter (Figure 10A–I). The restriction enzyme sites sequences of *Sac I* and *Hind III* were added with sequences of primer pairs (Table S1). PCR amplicons were cloned into t vector pMD 19 (simple) (Takara, Japan) and digested with *Sac I* and *Hind III* restriction enzymes (Takara, Japan). The DNA was extracted from the gel with a gel extraction kit ((Omega Bio-tek, Norcross, GA, USA) and ligated through T4 ligation (Takara, Japan) into pGL3 basic (luciferase reporter vector). Ligated pGL3 basic vector with inserts of the target fragments were then cloned into DH5α (Takara, Japan). Five single colonies were selected for each fragment and then sequenced (Sangon, Shanghai, China). Each fragment plasmid was extracted by Endo free Plasmid DNA Mini Kit II ((Omega Bio-tek, Norcross, GA, USA). Concentration of the extracted plasmid DNA was measured through Nano Quant plate TM (Infinite M200 PRO, TECAN, Switzerland) and stored at −20 °C.

### 4.11. Cell Culture and Transient Transfection

Bovine preadipocyte cell culture was maintained in Dulbecco’s Modified Eagle Medium (DMEM-F/12) (GIBCO, NY, USA) containing 10% FBS (Invitrogen, Waltham, MA, USA), 1% antibiotics (100 IU/mL penicillin and 100 μg/mL streptomycin) and incubated at 37 °C with 5% CO_2_. The cells were plated in 24-wells plates and transiently transfected when 70% to 90% of the confluence reached a density of 1.2 × 10^5^ cells with growth medium without antibiotics and FBS. The cultured cells were transfected with different fragments of plasmid DNA inserted with the *TORC2* promoter fragments, using lipofectamine 3000 transfection reagent (Invitrogen, Waltham, MA, USA) with pRL TK plasmid as an internal control vector to standardize transfection efficiency [81,82]. Forty-eight hours post transfection, cells were harvested and the lysate was subjected to luciferase assay for the measurement of relative transcriptional activities of TORC2-F1 to TORC2-F7 promoter fragments using dual-luciferase reporter assay System (Promega, Madison, WI, USA) as per the manufacturer’s protocol. The activities of both the renilla and firefly luciferase were measured using Nano Quant PlateTM (Infinite M200PRO, TECAN, Switzerland). The experiments were conducted in parallel and in triplicate.

### 4.12. Mutagenesis in Transcription Factor Binding Sites

The core sequence of the putative transcription factor binding sites for *C/EBPγ, XBP1, INSM1* and *ZNF263* motifs were mutated through site directed mutagenesis with the overlapping pairs of primers (Table S2) using the fast mutagenesis kit (TRANS bionovo, China).

### 4.13. C/BEPγ, XBP1, ZNF263 and INSM1 Knockdown

The transcription factors *C/BEPγ*, *XBP1*, *ZNF263* and *INSM1* were down-regulated through small interference RNA (siRNA). The details of the sequences have been mentioned in Table S1. The siRNAs against each transcription factors were commercially synthesized through GenePharma, Shanghai, China. The transcription and interference efficiency of each siRNA was evaluated through the expression regulation of respective transcription factor gene by qPCR analysis using gene specific primers (Table S1). Bovine preadipocyte were seeded in six-well culture plates and transiently transfected as mentioned above. 

### 4.14. Western Blot Analysis

The Qinchuan cattle preadipocyte cells were lysed with RIPA lysis buffer (Beyotime, Shanghai, China) which contains protease inhibitor Cocktail (Roche, Basel, Switzerland) for the extraction of total protein. The cellular protein was then mixed with protein loading buffer. Equal volumes of each protein samples were loaded into a 12% SDS-PAGE gel for electrophoresis. The band from the gel was then transferred to a nitrocellulose membrane (PVDF), blocked with 5% (w/v) skim milk through incubation at room temperature for 2 h. After blocking, the membrane was incubated with primary antibodies of anti-TORC2 rabbit polyclonal antibody (Sangon, Shanghai, China) and β-actin antibody (Abcam, Cambridge, UK) overnight. The IgG-Goat anti-Rabbit HRP antibody (1:2000, Abcam, Cambridge, UK) was used as a secondary antibody. Finally, chemical luminescence signals were identified by exposing the membrane photographed using Chemi Doc System (Bio-Rad, Hercules, CA, USA)

### 4.15. Cell Differentiation and Oil Red O Staining

Bovine preadipocyte cells were cultured, maintained and transfected with siRNAs as described earlier. After 48 h of transfection, the cells were induced to differentiation, with first differentiation media supplemented with 0.5 mM hydro cortisol, IBMX (3. isobut-1-methylxanthine), dexamethasone 1 μM, and insulin 167 nM as described previously (Kopp et al., 2014). Two days later, the media was changed to second differentiation medium containing DMEM-F/12 with 10% fetal bovine serum and insulin 167 nM. The cells were stained with Oil red O staining at day 9 of differentiation. Briefly, the stock solution for Oil red O was prepared by dissolving the staining powder in isopropanol under dark. Stock solution was filtered to prepare a working solution, diluted with 60% deionized water, and then filtered again. First, the cells were washed for three times with 1XPBS and then fixed with 4% paraformaldehyde solution for 30 min, at RT. After fixation, the cells were washed again three times with 1XPBS, and then, Oil red O staining (working solution) was applied and placed for 30 min at RT. Cells images were captured using the Olympus IX71 microscope (OLYMPUS, Tokyo, Japan) and lipid droplets were observed. Lastly, the stain was diluted with 100% isopropanol and lipids droplets were quantified through absorbance at 500 nm.

### 4.16. EMSAs (Electrophoretic Mobility Shift Assays)

Nuclear proteins were extracted from the preadipocyte cells of Qinchuan cattle through Nuclear Extraction Kit (Active Motif Corp, Carlsbad, CA, USA) and stored at −80 °C. The 5′-biotinated, un-biotinated and site directed mutated probes for *C/EBPγ, XBP1, INSM1* and *ZNF263* transcription factor motifs were synthesized (Table S1) (Sangon, Shanghai, China). The Electrophoretic Mobility Shift Assays for each transcription factor were performed using Lightshift Chemiluminescent EMSA Kit (Thermo Fisher Corp., Waltham, MA, USA). In summary, 200 fmol of the 5′-biotinated probes were incubated with 10× binding buffer 2 µL, poly dl.dc 1 µL, 50% glycerol 1 µL and mixed with 10 µg of nuclear protein to make a total volume of 20 µL reaction mixture. In slight modification, we added specific competitor (non-biotinated) and non-specific competitor (mutated) probes into the reaction mixture. To perform super shift assay, anti-C/EBPγ (LS-C485400, LS-bio, Shanghai, China), anti-XBP1 (ab220783, Abcam, Cambridge, UK), anti-INSM1 and anti-ZNF263 (Sangon, Shanghai, China) antibodies were mixed with nuclear protein and incubated for 30 min on ice, then, the reagent mixture was added to the antibodies, and nuclear protein complex and incubated for 15 min at 37 °C. The biotin labeled probes were added and incubated for 20 min on ice. The DNA-protein complex was separated through 6% non-denaturing polyacrylamide gel through PAGE polyacrylamide electrophoresis system within 0.5% XTBE buffer solution. The image was captured through Chemi Doc-XRS imager-system (Bio-Rad, Hercules, CA, USA).

### 4.17. Statistical Analysis

The data are presented as mean ± SEM. SPSS 20.0 software was used for statistical analysis and variation between the groups was determined through the Student’s t-test. Variations were considered statistically significant at * *p* < 0.05, *** *p* < 0.01, and **** *p* < 0.001.

## 5. Conclusions

We can conclude from the present study that *TORC2* positively regulates both bovine adipocyte proliferation and differentiation. Moreover, we identified the core promoter region of *TORC2* gene spanning from −314 to −69 bp upstream of transcription start site. In addition to that, we identified that *C/EBPγ*, *XBP1*, *INSM1* and *ZNF263* regulate *TORC2* gene as activators transcription factors in the promoter of the *TORC2* gene as well. These findings will not only provide an insight for the improvement of intramuscular fat in cattle, but will additionally provide a clue to intervene the issue of metabolic syndrome and obesity in human beings.

## Supplementary Materials

Supplementary materials can be found at https://www.mdpi.com/1422-0067/20/18/4338/s1.
