# Supplementary material for: Function and Transcriptional Regulation of Bovine TORC2 Gene in Adipocytes: Roles of C/EBPγ, XBP1, INSM1 and ZNF263
Source: Int J Mol Sci. 2019 Sep 4;20(18):4338. doi: 10.3390/ijms20184338 (PMC6769628; doi:10.3390/ijms20184338)
Supplement: Supplementary file 1 [file ijms-20-04338-s001.pdf]

Table S1. Primers used in this paper

Primer Name	Purpose	Primer sequence	Tm (^{0}C)	Product length (bp)
**Forward Fragment 1**	Luciferase assay	gagctcGACACGAGCTCAGTCAG	62.40	1990
**Forward Fragment 2**	Luciferase assay	gagctcCCAAATCTAGAAAGCTCCAAGACAG	62.12	1690
**Forward Fragment 3**	Luciferase assay	gagctcCGGTTACTTGTAAGGCTGGAGGT	65.68	1370
**Forward Fragment 4**	Luciferase assay	gagctcCCCTGGCCAGCACATCAGTGGAA	70.45	1047
**Forward Fragment 5**	Luciferase assay	gagctcCACCCCCAGGGTTATTTTTG	62.41	801
**Forward Fragment 6**	Luciferase assay	gagctcCTTCCCTCCCTGTCTCCACCCCT	71.24	504
**Forward Fragment 7**	Luciferase assay	gagctcCAAACTACAATTCCCGGCGATC	63.66	259
**Reverse TORC2**	Luciferase assay	aagcttGTGTCAAGCTTCAGCGCGATCTTCTCA	66.25	For all fragments
**C/EBPγ-Bos-225 F**	Interference	CCGGGCGUGAACGGAAUAATT		
**C/EBPγ-Bos-225 R**	Interference	UUAUUCCGUUCACGCCCGGTT		
**C/EBPγ-Bos-358 F**	Interference	GCUCACCCAUGGAUCGCAATT		
**C/EBPγ-Bos-358 R**	Interference	UUGCGAUCCAUGGGUGAGCTT		
**ZNF263-Bos-416 F**	Interference	GCUUCAGACGGUUCCGCUUTT		
**ZNF263-Bos-416 F**	Interference	AAGCGGAACCGUCUGAAGCTT		
**ZNF263-Bos-639 F**	Interference	GGAUAUGCAGAAAGAACUUTT		
**ZNF263-Bos-639 F**	Interference	AAGUUCUUUCUGCAUAUCCTT		
**INSM1-Bos-933 F**	Interference	GCUCAAGAUCAAGGAGGGUTT		
**INSM1-Bos-933 R**	Interference	ACCCUCCUUGAUCUUGAGCTT		
**INSM1-Bos-1424 F**	Interference	CCGAGGACCUACUGGCCUUTT		
**INSM1-Bos-1424 R**	Interference	AAGGCCAGUAGGUCCUCGGTT		
**XBP1-Bos-337 F**	Interference	GGAGCAACAAGUGGUAGAUTT		
**XBP1-Bos-337 R**	Interference	AUCUACCACUUGUUGCUCCTT		
**XBP1-Bos-460 F**	Interference	GGAUGCCCUAGUGACUGAATT		
**XBP1-Bos-460 R**	Interference	UUCAGUCACUAGGGCAUCCTT		
**XBP1 F**	Mutation	GGCTGTTAAAGAGTCGTACAAACAAACTA	56.48	500
**XBP1 R**	Mutation	GTACGACTCTTTAACAGCCCCAGTCACGC	64.96	500
**C/EBPγ F**	Mutation	GTTTCCTCTCTCGTAGGTCGTGACTGGGG	65.94	500
**C/EBPγ R**	Mutation	GACCTACGAGAGAGGAAACGGACACCATT	65.94	500
**INSM1 F**	Mutation	TCGTATTGCGTGACTATCTCTGTTAAAGA	56.69	500
**INSM1 R**	Mutation	AGATAGTCACGCAATACGAGAGAGGAAAC	59.43	500
**ZNF263 F**	Mutation	GGGAGGAACCAACAGCATGGAGCAAAATC	64.04	500
**ZNF263 R**	Mutation	CATGCTGTTGGTTCCTCCCTGCGTGGAGG	69.48	500
**C/EBPγ F non- biotinated**	Probe	CCGTTTCCTCTCTCGTATTGCGTGACTGGGGCTC	69.84	
**C/EBPγ R non- biotinated**	Probe	GAGCCCCAGTCACGCAATACGAGAGAGGAAACGG	69.84	
**C/EBPγ F Mutated**	Probe	CCGTTTCCTCTCTCGTAGGTCGTGACTGGGGCTC	70.89	
**C/EBPγ R Mutated**	Probe	GAGCCCCAGTCACGACCTACGAGAGAGGAAACGG	70.89	
**XBP1 F non- biotinated**	Probe	GGGGCTGTTAAAGAGTCACGTAAACAAACTACAA	61.23	
**XBP1 R non- biotinated**	Probe	TTGTAGTTTGTTTACGTGACTCTTTAACAGCCCC	61.23	
**XBP1 F Mutatated**	Probe	GGGGCTGTTAAAGAGTCGTACAAACAAACTACAA	61.23	
**XBP1 R Mutatated**	Probe	TTGTAGTTTGTTTGTACGACTCTTTAACAGCCCC	61.23	
**INSM1 F non- biotinated**	Probe	TCTCGTATTGCGTGACTGGGGCTGTTAAAGAGTC	65.76	
**INSM1 R non- biotinated**	Probe	GACTCTTTAACAGCCCCAGTCACGCAATACGAGA	65.76	
**INSM1 F Mutated**	Probe	TCTCGTATTGCGTGACTATCTCTGTTAAAGAGTC	59.97	
**INSM1 R Mutated**	Probe	GACTCTTTAACAGAGATAGTCACGCAATACGAGA	59.97	
**ZNF263 F non- biotinated**	Probe	CAGGGAGGAACCAACAGGGAGGAGCAAAATCCCG	70.35	
**ZNF263 R non- biotinated**	Probe	CGGGATTTTGCTCCTCCCTGTTGGTTCCTCCCTG	70.35	
**ZNF263 F Mutated**	Probe	CAGGGAGGAACCAACAGCATGGAGCAAAATCCCG	69.20	
**ZNF263 R Mutated**	Probe	CGGGATTTTGCTCCATGCTGTTGGTTCCTCCCTG	69.20	
**C/EBPγ F biotinated**	Probe	CCGTTTCCTCTCTCGTATTGCGTGACTGGGGCTC	69.84	
**C/EBPγ R biotinated**	Probe	GAGCCCCAGTCACGCAATACGAGAGAGGAAACGG	69.84	
**XBP1 F biotinated**	Probe	GGGGCTGTTAAAGAGTCACGTAAACAAACTACAA	61.23	
**XBP1 R biotinated**	Probe	TTGTAGTTTGTTTACGTGACTCTTTAACAGCCCC	61.23	
**INSM1 F biotinated**	Probe	TCTCGTATTGCGTGACTGGGGCTGTTAAAGAGTC	65.76	
**INSM1 R biotinated**	Probe	GACTCTTTAACAGCCCCAGTCACGCAATACGAGA	65.76	
**ZNF263 F biotinated**	Probe	CAGGGAGGAACCAACAGGGAGGAGCAAAATCCCG	70.35	
**ZNF263 R biotinated**	Probe	CGGGATTTTGCTCCTCCCTGTTGGTTCCTCCCTG	70.35	
**R1-RACE *TORC2***	Probe	CCCTCACCCGGGTGGAGCCGATGTC	78	266
**R2-RACE *TORC2***	Probe	GCGTTCCGCCTCTTTCGCTACGGCT	75	359
**TORC2 F**	Expression	GAGGAGGTGATGATGGAC	52.05	138
**TORC2 R**	Expression	GCTCTGGAACTCGGCTAG	55.57	138
**C/EBPγ F**	Expression	CTGGAGGGGCCAGGTACA	60.22	

**C/EBPγ R**	Expression	ATACTCAGGCTCATGCCAGC	57.62	
**XBP1 F**	Expression	AAGCCAAAGTAGAGGGGGTGTG	59.71	
**XBP1 R**	Expression	CTGGGATGCTGAGAAGGGAAAA	57.23	
**INSM1 F**	Expression	CACATCAACAAGTGCCACCC	57.22	
**INSM1 R**	Expression	AGCGGAGACCACACTTTCAC	57.80	
**ZNF263 F**	Expression	GCAAAGGATTAGCATAAAACTGAAAA	51.41	
**ZNF263 R**	Expression	AGGACAGAATAAGGCACCCACA	58.38	
**GAPDH F**	Expression	AGTTCAACGGCACAGTCAAGG	58.09	124
**GAPDH R**	Expression	ACCACATACTCAGCACCAGCA	58.98	124
**CDK1 F**	Expression	AGTGGAAACCAGGAAGCTTAG		
**CDK1 R**	Expression	ATTCGTTTGGCAGGATCATAGA		
**CDK2 F**	Expression	GGGTCCCTGTTCGTACTTATAC		
**CDK2 R**	Expression	CCACTGCTGTGGAGTAGTATTT		
**PCNA F**	Expression	CCTTGGTGCAGCTAACCCTT		
**PCNA R**	Expression	TTGGACATGCTGGTGAGGTT		
**SREBP-1 F**	Expression	CAATGTGTGAGAAGGCCAGT		
**SREBP-1 R**	Expression	ACAAGGAGCAGGTCACACAG		
**ACCα F**	Expression	CTCCAACCTCAACCACTACGG		
**ACCα R**	Expression	GGGGAATCACAGAAGCAGCC		
**PPARγ F**	Expression	GAGATCACAGAGTACGCCAAG		
**PPARγ R**	Expression	GGGCTCCATAAAGTCACCAA		
**CEBPα F**	Expression	ATCTGCGAACACGAGACG		
**CEBPα R**	Expression	CCAGGAACTCGTCGTTGAA		
**ELOVL6 F**	Expression	CGTAGCGACTCCGAAGATCA		
**ELOVL6 R**	Expression	ACAGGGCGGAAAACAGGAAA		
**PLIN2 F**	Expression	CTGTCTACCAAGCTCTGCTC		
**PLIN2 R**	Expression	CGATGCTTCTCTTCCACTCC		
**P27 F**	Expression	AGATGTCAAACGTGCGAGTG		
**P27 R**	Expression	GCCAAAGAGGTTTCTGCAAG		
**P21 F**	Expression	GACCAGCATGACAGATTTCTACCA		
**P21 R**	Expression	TGAAGGCCCAAGGCAAAAG		
**Mcm6 F**	Expression	TCTTCATGGAGGATTACAGTGCG		
**Mcm6 R**	Expression	CGAGATTGACATCAGGTGTTTCC		
**TORC2 F**	CDS cloning	GTTTAAACTTAAGCTTATGGCGACGTCGGGAGCG		
**TORC2 R**	CDS cloning	GCCCTCTAGACTCGAGTCACTGCAGCCGGTCACTG		

Table S2. Comparative transcriptional variation of different fragments with activity of pGL3-basic vector analyzed through Tukey's multiple comparison test.

Fragments comparisons	Mean variation	95% CI of diff.	Significance	Summary
**PGL3-Basic vs. -69/+190**	-10.45	-36.64 to 15.75	No	Ns
**PGL3-Basic vs. -314/+190**	-71.54	-97.73 to -45.34	Yes	****
**PGL3-Basic vs. -611/+190**	-81.60	-107.8 to -55.40	Yes	****
**PGL3-Basic vs. -857/+190**	-31.65	-57.85 to -5.458	Yes	*
**PGL3-Basic vs. -1180/+190**	-49.57	-75.76 to -23.37	Yes	***
**PGL3-Basic vs. -1500/+190**	-32.07	-58.26 to -5.871	Yes	*
**PGL3-Basic vs. -1800/+190**	-44.60	-70.79 to -18.40	Yes	***
